# Inferring the potential risks of H7N9 infection by spatiotemporally characterizing bird migration and poultry distribution in eastern China

**DOI:** 10.1186/2049-9957-2-8

**Published:** 2013-05-03

**Authors:** Benyun Shi, Shang Xia, Guo-Jing Yang, Xiao-Nong Zhou, Jiming Liu

**Affiliations:** 1 Department of Computer Science, Hong Kong Baptist University, Waterloo Road, Kowloon Tong, Hong Kong; 2 The Jockey Club School of Public Health and Primary Care, Chinese University of Hong Kong, Hong Kong; 3 National Institute of Parasitic Diseases, Chinese Center for Disease Control and Prevention, Shanghai 200025, China; 4 Key Laboratory of Parasite and Vector Biology, MOH, Shanghai 200025, China; 5 WHO Collaborating Center for Malaria, Schistosomiasis and Filariasis, Shanghai 200025, China

## Abstract

**Background:**

In view of the rapid geographic spread and the increasing number of confirmed cases of novel influenza A(H7N9) virus infections in eastern China, we developed a diffusion model to spatiotemporally characterize the impacts of bird migration and poultry distribution on the geographic spread of H7N9 infection.

**Methods:**

Three types of infection risks were estimated for 12 weeks, from February 4 to April 28, 2013, including (i) the risk caused by bird migration, (ii) the risk caused by poultry distribution, and (iii) the integrated risk caused by both bird migration and poultry distribution. To achieve this, we first developed a method for estimating the likelihood of bird migration based on available environmental and meteorological data. Then, we adopted a computational mobility model to estimate poultry distribution based on annual poultry production and consumption of each province/municipality. Finally, the spatiotemporal risk maps were created based on the integrated impacts of both bird migration and poultry distribution.

**Results:**

In the study of risk estimation caused by bird migration, the likelihood matrix was estimated based on the 7-day temperature, from February 4 to April 28, 2013. It was found the estimated migrant birds mainly appear in the southeastern provinces of Zhejiang, Shanghai and Jiangsu during Weeks 1 to 4, and Week 6, followed by appearing in central eastern provinces of Shandong, Hebei, Beijing, and Tianjin during Weeks 7 to 9, and finally in northeastern provinces of Liaoning, Jilin, and Heilongjiang during Weeks 10 to 12.

In the study of risk caused by poultry distribution, poultry distribution matrix was created to show the probability of poultry distribution. In spite of the fact that the majority of the initial infections were reported in Shanghai and Jiangsu, the relative risk of H7N9 infection estimated based on the poultry distribution model predicted that Jiangsu may have a slightly higher likelihood of H7N9 infection than those in Zhejiang and Shanghai, if we only take the probability of poultry distribution into consideration.

In the study of integrated risk caused by both bird migration and poultry distribution, the higher risk in southeastern provinces occurred during the first 8 weeks, and that in central eastern provinces appeared during Weeks 8 to 12, and that in northeastern provinces since Week 12. Therefore, it is necessary to regulate the poultry markets as long as the poultry-to-poultry transmission is not so well understood.

**Conclusion:**

With reference to the reported infection cases, the demonstrated risk mapping results will provide guidance in active surveillance and control of human H7N9 infections by taking intensive intervention in poultry markets.

## Multilingual abstracts

Please see Additional file 1 for translations of the abstract into the six official working languages of the United Nations.

## Background

The emergence of a novel influenza A(H7N9) virus have drawn the global concerns about the possibility of a new influenza pandemic in human society [1,2]. Until April 22, 2013, the number of confirmed H7N9 bird flu cases in China has increased to 104, resulting in 21 deaths [3]. The confirmed infection cases are concentrated in eastern China, including Shanghai, Zhejiang, Jiangsu, Anhui, Henan, and Beijing. The Chinese health authorities have reported that avian influenza A(H7N9) was detected from the samples of chickens and ducks, as well as samples from live bird markets. Meanwhile, researchers have pointed out that the outbreak may be caused by the migration of wild birds, e.g., waterfowl [4,5]. However, the investigation of the source and the mode of H7N9 transmission is still in its infancy due to the lack of solid evidence and surveillance data.

Due to the rapid geographic spread and the increase of confirmed cases, it would be necessary and desirable to estimate the risks of H7N9 infection in different provinces/municipalities of eastern China. However, at present it remains quite challenging to build comprehensive transmission models for H7N9 virus because the mode of transmission has not yet been well understood. For example, based on a report by European Certre for Disease Prevention and Control on April 19, 2013, there is no evidence of sustained human-to-human transmission. Moreover, based on an official statement from the Chinese Ministry of Agriculture, only 39 samples are positive for the H7N9 virus among 47,801 tests samples taken from live animal markets, farms, and slaughter houses. This implies that there is even no convincing evidence of sustained poultry-to-poultry transmission. Therefore, in this paper, we consider the possibility that infected birds/poultry in one region cause infections in another due to bird migration and/or poultry distribution. Specifically, we use the likelihood of bird migrating to (or the relative quantity of poultry being distributed to) certain provinces/municipalities to infer their risks of human infection.

In this paper, we present a diffusion model to infer the geographic risks of H7N9 infection by spatiotemporally characterizing both bird migration in eastern China and poultry distribution in mainland China. Specifically, we pay attention to investigating three types of risk: (i) the risk caused by bird migration, (ii) the risk caused by poultry distribution, and (iii) the integrated risk caused by both bird migration and poultry distribution. First, to determine the impacts of bird migration, we develop a method for estimating the likelihood of bird migration from one location to another. Then, to quantify the impacts of poultry distribution on H7N9 spread, we adopt a computational mobility model to estimate the poultry distribution among provinces/municipalities in mainland China based on their annual poultry production and consumption volumes. Accordingly, for each type of infection risk, we aim to infer the potential spatiotemporal patterns of H7N9 infection. Finally, we discuss the limitations and potential extensions of our models in more complicated transmission situations.

## Methods

### Data sources

In order to characterize bird migration from south regions to north regions during spring season, we consider 12 provinces that belong to bird migration routes of eastern China [6]. In the sequence from south to north, they are Zhejiang (ZJ), Shanghai (SH), Jiangsu (JS), Anhui (AH), Henan (HN), Shandong (SD), Hebei (HB), Tianjin (TJ), Beijing (BJ), Liaoning (LN), Jilin (JL), and Heilongjiang (HLJ). Here, we propose a computational method by taking into consideration three environmental/meteorological factors that may affect the route of bird migration: (i) wetland area, (ii) temperature, and (iii) traveling distance. Specifically, we collect the data of wetlands in each of the above-mentioned regions from the China Statistical Yearbook 2012 [7]. Further, we compute the proportion of wetlands area to the total area of each province. Specifically, to concentrate on our interested questions, we simplify each province as a node in our model. In doing so, we calibrate the traveling distance in the context of bird migration as the spatial distance between the capitals of two provinces. Finally, we collect the historical temperature data in the capital of each province during the period between February 4, 2013 and April 28, 2013 (i.e., 12 weeks) [8]. To achieve more accurate estimation in the future, more detailed data from each province should be collected.

To estimate the poultry distribution in mainland China, we collect the data on the annual poultry production and consumption of each province/municipality from China Animal Husbandry Yearbook 2012 and China Statistical Yearbook 2012 [7].

### Characterizing geographic spread of H7N9 Infection

In this paper, we pay special attention to the geographic spreading of H7N9 infection in eastern China. Because at the present time there is no convincing evidence of sustained human-to-human or poultry-to-poultry transmission, we do not attempt to build a comprehensive model for H7N9 transmission. Nevertheless, we focus on inferring the risk of H7N9 infection in different locations by characterizing the impacts of bird migration and poultry distribution from one location to another. Our model is based on the concept of reproduction matrix ***K***:

(1) $$\dot{I}=\mathrm{KI}$$

where ***I*** represents the number of infections, and ***K*** characterizes the migration of birds and/or distribution of poultry in mainland China. Note that in Wallinga et al’s study [9], the reproduction matrix ***K*** is known as the “next generation matrix” for deterministic models [10], and “mean offspring matrix” for stochastic models [11].

The estimation of ***K*** is twofold. First, the poultry distribution matrix among 31 provinces/municipalities are deterministic, which is estimated based on statistical data about poultry production and consumption in each province/municipality. Second, bird migration is dependent on many time-varying factors (e.g., temperature). Therefore, we treat bird migration as a stochastic process to calculate the likelihood of bird migration. Accordingly, we investigate three types of potential risk caused by either bird migration, poultry distribution, or both of them.

### Estimating bird migration in eastern China

In view of the lack of accurate data for characterizing bird migration along the migration routes in eastern China, we adopt a computational approach to inferring the likelihood of bird migrating among different regions from the available environmental and meteorological data.

In what follows, we use *N* to denote the total number of considered provinces and *i* or *j* to represent the index of each province. During the spring season (i.e., February, March, and April), birds in eastern China mainly migrate from south to north. Here, we use *m*_*ij*_ to describe the likelihood of bird migration between two provinces *i* and *j*. In order to estimate *m*_*ij*_, we consider three factors that may affect bird migration: (i) wetland area *L*_*i*_, (ii) traveling distance *d*_*ij*_, and (iii) temperature *T*_*i*_. Specifically, we consider the likelihood for bird migration between two provinces are proportional to the area of wetlands, and inversely proportional to the traveling distance. In addition, bird migration occurs in certain regions with suitable temperature $\tilde{T}$.

Analogous to the gravity law of mobility patterns [12,13], we compute the likelihood of bird migration *m*_*ij*_ between provinces *i* and *j* as follows:

(2) $$m_{\text{ij}}=\mu \frac{L_{i}L_{j}}{d_{\text{ij}}^{2}}\cdot \frac{1}{\text{Var}(T_{i},T_{j})}$$

and

(3) $$\text{Var}(T_{i},T_{j})={\left({\left(T_{i}-\tilde{T}\right)}^{2}+{\left(T_{i}-\tilde{T}\right)}^{2}\right)}^{\frac{1}{2}}$$

where *Var*(*T*_*i*_,*T*_*j*_) describes the variance between the temperatures *T*_*i*_ and *T*_*j*_ with suitable temperature $\tilde{T}$, and *μ* is a scaling constant. The matrix ***M*** with the elements of *m*_*ij*_ is the overall likelihood matrix that describes the probability of bird migration among different regions.

### Estimating poultry distribution in mainland China

We estimate the poultry distribution among different provinces/municipalities based on their annual production and consumption. First, we normalize the production *s*_*i*_ and consumption *c*_*i*_ of each province/municipality *i*, i.e., $s_{i}=s_{i}/\underset{i}{\sum }s_{i}$ and $c_{i}=c_{i}/\underset{i}{\sum }c_{i}$. Then, based on Simini et al’s study [14], we treat the normalized consumption of each province/municipality as an “attractor”. Next, for each province/municipality with extra production (i.e., {*i*∈*S*|*s*_*i*_−*c*_*i*_>0}), we calculate the probability of one unit of poultry flowing from *i* to any *j*∉*S* as follows:

(4) $$p_{\text{ij}}=\frac{c_{i}c_{j}}{\left(c_{i}+c_{\text{ij}})(c_{i}+c_{j}+c_{\text{ij}}\right)}$$

where *c*_*i*_ and *c*_*j*_ represent the normalized consumption of poultry in locations *i* and *j*, respectively; *c*_*ij*_ refers to the total normalized consumption in locations within the circle of radius *r*_*ij*_ certred at *i* (excluding the consumption in *i* and *j*). Here, *r*_*ij*_ is the adjacent distance of different provinces/municipalities in mainland China. Then, we calculate the relative impacts *w*_*i*_ of each *i*∈*S* to others as follows:

(5) $$w_{i}=\frac{s_{i}-c_{i}}{\underset{i}{\sum }(s_{i}-c_{i})}.$$

Based on the definition of reproduction matrix ***K***, each element *k*_*ij*_∈***K*** can be calculated as *k*_*ij*_=*w*_*j*_*p*_*ji*_.

### Integrating the impacts from both bird migration and poultry distribution

There are two steps to integrate the impacts from both bird migration and poultry distribution on each province/municipality. First, based on the estimated bird migration matrix, we calculate the probability of migratory birds that will be in a specific region during a specific time window (i.e., one week in this study). Specifically, the probability of birds located in *i* at time window *t*−1 migrating to *j* at time window *t* can be calculated as follows:

(6) $$\sigma_{\text{ij}}(t)=\underset{k}{\sum }m_{\text{ik}}(t-1)\cdot m_{\text{kj}}(t).$$

Second, after calculating the integrated probability of bird migrating from different regions (i.e., $\underset{i}{\sum }\sigma_{\text{ij}}(t)$), we further consider the impacts of poultry distribution. In this case, the integrated impacts of *j* to *l* at time window *t* can be calculated as follows:

(7) $$\underset{i}{\sum }\sigma_{\text{ij}}(t)\cdot k_{\text{lj}}.$$

## Results

### The infection risk from bird migration in eastern China

**Figure 1:**
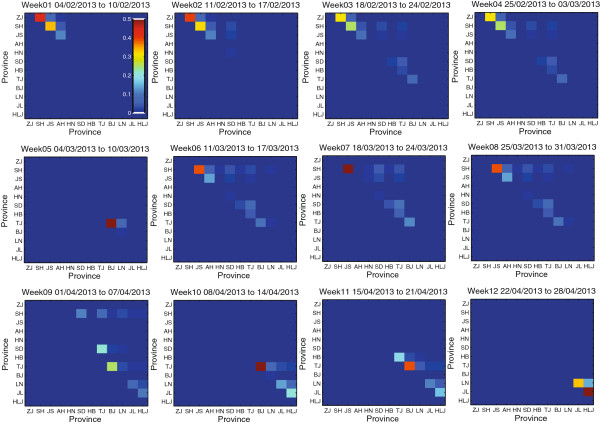
**The likelihood of bird migration from one province or municipality to another.** The estimated likelihood of bird migration from one province or municipality to another from February 4 to April 28, 2013 with 7 days as a time window. The likelihood matrix for Week 12 is calculated based on 7-day temperature prediction on April 22, 2013.

**Figure 2:**
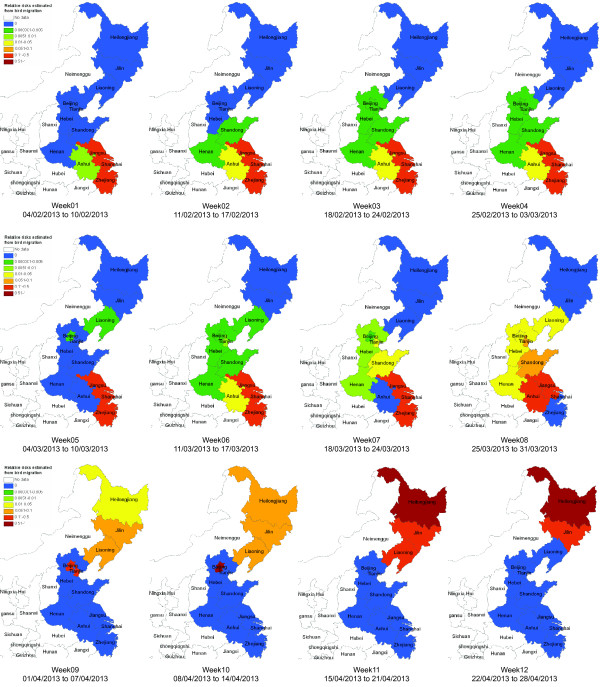
**The estimated spatiotemporal patterns of infection risks based on the likelihood of bird migration.** The estimated spatiotemporal patterns of infection risks based on the likelihood of bird migration from February 4 to April 28, 2013. It can be observed that after Week 7, the birds gradually migrate from southern provinces, to central and northeastern provinces.

To estimate the effects of bird migration on the geographic spread of H7N9 infections, we first calculate the likelihood of bird migration from one province/municipality to another in eastern China. Based on our model, we consider the suitable temperature $\tilde{T}$ as the average temperature (i.e., 6 centigrade) in Shanghai from February 12 to 19, 2013, which was one week before the first case of H7N9 infection was reported. Figure 1 shows the results of bird migration likelihood from February 4 to April 28, 2013, with 7 days as a time window “Week”. Note that likelihood matrix for Week 12 is calculated based on the 7-day temperature prediction on April 22, 2013. It can be observed that the estimated migratory birds during the Weeks 1, 2, 3, 4, and 6 mainly appear in the provinces of Zhejiang, Shanghai, and Jiangsu. Then, during the Weeks 7, 8, and 9, the birds are moving from eastern provinces to central provinces (i.e., Shandong, Hebei, Beijing, and Tianjin). Finally, as for the Weeks 10, 11, and 12, the most migratory birds move to northeastern provinces of China (i.e., Liaoning, Jilin, and Heilongjiang). Based on the likelihood of bird migration in eastern China, we can further estimate its impacts on the spatiotemporal spread of H7N9 infection. Since it is reported that the migratory birds would have nested in Yangtze River Delta region until late March, we assume that the birds will constantly migrate based on the estimated likelihood at the first 8 weeks. Figure 2 shows the estimated spatiotemporal bird migration from February 4 to April 28, 2013, which may indirectly reflect the human infection risk. It can be observed that after Week 7, the birds migrate from southern provinces, to central provinces, and finally to northeastern provinces. Note that the colors shown in the figures only reflect the relative risks of the 12 provinces/municipalities in a specific week.

The estimated spatiotemporal patterns of bird migration be well matched with the observations of the public news released by local media, reporting the appearance of migratory birds in their regions. For example, researchers reported that the migratory birds nesting in Yangtze River Delta region (i.e., Shanghai, Jiangsu, and Zhejiang) would leave for north in earlier February until mid/late March^a^. In late March and early April, Jiangsu and Hebei reported their observations of migratory birds^b^. Consequently, Beijing and Tianjin experienced the bird migration peaks^c^. In mid April, the cities located in northeastern China (i.e., Shenyang and Dalian in Liaoning province, and Jiamusi in Heilongjiang province) reported their observations of bird migration. And predicted the peak would appear in late April^d^.

### The infection risk from poultry distribution in mainland China

**Figure 3:**
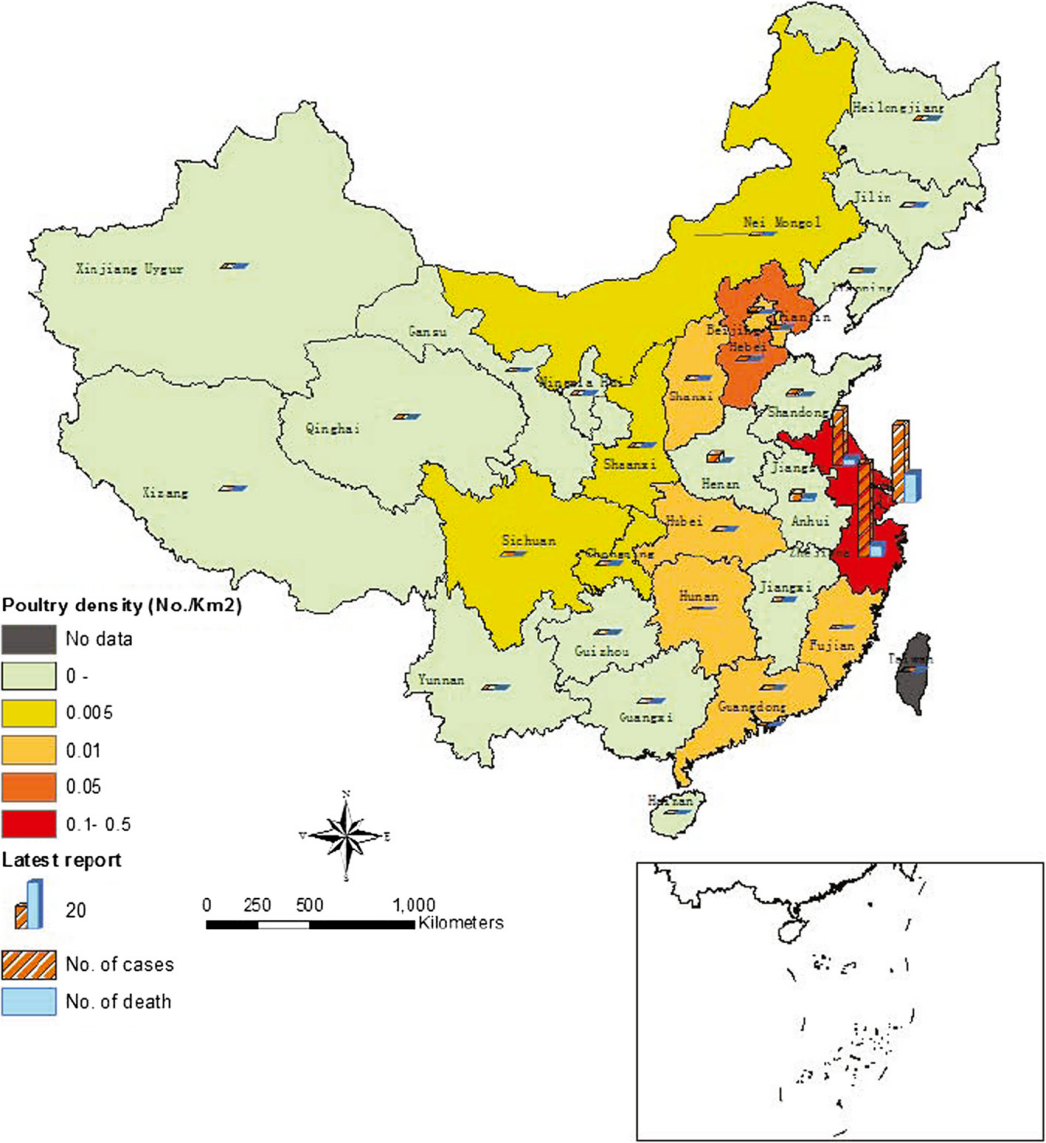
**The map of relative H7N9 infection risk in mainland China.** The map of relative H7N9 infection risk in mainland China estimated by assuming that the source of infection originated from the southeastern regions of Zhejiang, Shanghai, and Jiangsu (Jiangsu has a slightly higher likelihood). We can find that if we only consider poultry distribution, Jiangsu have relatively larger impacts on H7N9 infection in Zhejiang and Shanghai, where most infection cases were reported. Meanwhile, we can also observe that Jiangsu can also affect the infection risks of other regions that are relatively far away from Jiangsu.

Based on the poultry production and consumption in 31 provinces/municipalities of mainland China, we can estimate the probability of poultry distribution from one province/municipality to another. Based on the estimation, we can further examine the impacts of each province/municipality on the geographical spread of H7N9 infection. In this paper, since the majority of the confirmed infections in the early stage were reported in Shanghai and Jiangsu province, we assume that virus may come from these two regions. Because Shanghai’s annual consumption is larger than its annual production, Shanghai does not distribute poultry to other regions. Therefore, we show the relative risks of H7N9 infection estimated only by poultry distribution, e.g., from Jiangsu (see Figure 3). We can find that Jiangsu can significantly affect H7N9 infection in Zhejiang and Shanghai, where most infection cases were reported. Meanwhile, we also observe that Jiangsu can also affect the infection risks of other regions that are relatively far away from Jiangsu. In this case, it would be necessary to regulate the poultry markets as long as the poultry-to-poultry transmission is still not well understood.

### The integrated infection risk from both bird migration and poultry distribution

**Figure 4:**
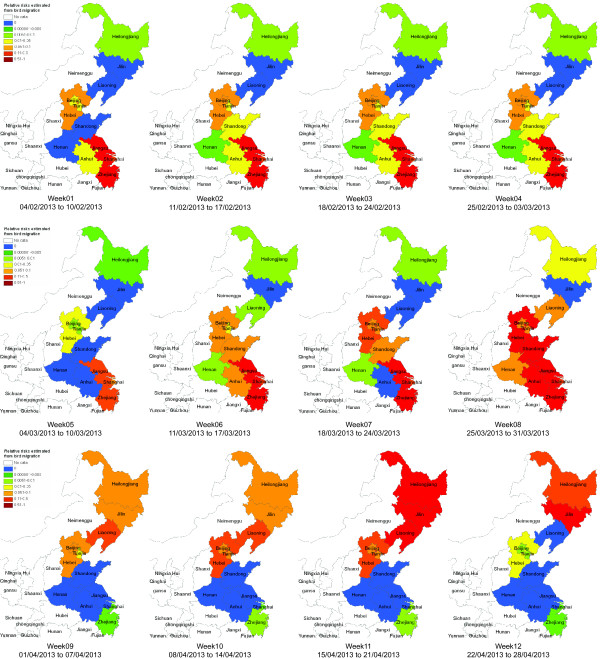
**The estimated spatiotemporal patterns of integrated risk caused by both bird migration and poultry distribution in eastern China.** The estimated integrated risk caused by both bird migration and poultry distribution in eastern China. It can be found that during the first 8 weeks, except for Jiangsu, Shanghai, and Zhejiang, other provinces such as Hebei, Anhui, Shangdong, and Beijing, also have the risk of H7N9 infection. After Week 8, different from the estimations in Figure 2, we can still observe sustained infection risks in Hebei, Tianjin, and Beijing until Week 12.

In reality, the geographical risks of H7N9 infection may be correlated with the integrated impacts of both bird migration and poultry distribution. Figure 4 shows the estimations of the integrated risk of each province/municipality in eastern China. Comparing with the results in Figure 2, we can find that during the first 8 weeks, except for Jiangsu, Shanghai, and Zhejiang, other provinces such as Hebei, Anhui, Shangdong, and Beijing, also have the risks of H7N9 infection. After Week 8, as the bird migrate from south to north, the provinces at risk also change accordingly. However, due to the impacts of poultry distribution, comparing with the estimations in Figure 2, we can still observe the sustained infection risks in Hebei, Tianjin, Beijing until Week 12. The results as shown in Week 12 are predicted based on our model for the estimated 7-day temperature on April 22, 2013. It can be found that during this week, Jilin and Heilongjiang could be the most risky provinces since most birds will migrate there. Similar to Figure 2, the colors in each week just show the relative risks of the 12 provinces/municipalities, which means that the potential risk of Shandong province in Week 8 is not necessarily severer than the potential risk in Week 7.

## Discussion

According to Wallinga et al’s study [9], for a large class of transmission models, the reproduction matrix ***K*** can be written as

(8) K=SABC.

Here, ***S*** is a diagonal matrix with the location-specific number of susceptible poultry (i.e., the supply of poultry in each location) on the diagonal; ***A*** is a diagonal matrix representing the per contact probability of acquiring infection at each location; ***B*** is a matrix referring to the poultry distribution and bird migration from one location to another; ***C*** is a diagonal matrix with the location-specific per contact of probability of transmitting infection on the diagonal. Further, if we do not know the epidemiological properties of H7N9 virus for poultry, we can assume that the diagonal values of matrices ***A*** (or ***C***) are identical, i.e., the acquisition probability of H7N9 and the force of infection are the same for the poultry at different locations. By doing so, we can study the stable state of the model after the transmission period is finished without considering human interventions. Moreover, we can also suggest appropriate intervention strategies based on the properties of the reproduction matrix [9,15].

At the present time, we still do not have enough information to build comprehensive transmission models for H7N9, for instance: (i) the source of the transmission has not yet been confirmed, (ii) the mode of transmission has not yet been well understood, and (iii) there is even no convincing evidence of sustained poultry-to-poultry transmission. Due to these limitations, in this paper, we only consider a diffusion model by assuming that there is no human-to-human and poultry-to-poultry transmission. Specifically, we use the geographic diffusion of birds or poultry (due to either migration or distribution) to infer the risk of H7N9 infection. In doing so, the estimated risk may not exactly reflect the confirmed cases of human infection. This is because human infections may depend on many other issues, such as the human movement from one region to another, and the mode of birds-to-poultry, poultry-to-human, and bird-to-human transmission. Moreover, the estimated potential risk in each region is earlier than the reported human infections. This is because it may take a period of time for an infected individual develop symptoms. Once more information is available, we are ready to address the above-mentioned limitations by designing more accurate models.

Up to time when this paper is written (on April 22), the number of cases of H7N9 infection is still increasing in eastern China. The source and the mode of transmission have not yet been confirmed. Some preliminary studies have suggested that influenza A(H7N9) virus may spread when humans contact with poultry population or poultry products (e.g., several report cases are correlated with live poultry markets). Experts have proposed that bird migrations during the spring season in eastern China may play a significant role in the emergence of infection cases in different regions. In view of these reasons, in this study, we have estimated the relative risks of influenza A(H7N9) virus by incorporating the information about both bird migration and poultry distribution in eastern China. Particularly, we produced the potential risk map based on the integration of bird migration and poultry distribution, at least three types of risk for human infection with influenza A(H7N9) virus (Figures 2, 3, and 4), which can serve as tools for active surveillance and response to the outbreaks of H7N9 infections. However, due to the lack of sufficient data at the present stage, we have only considered a computational approach to estimating the likelihood of bird migration in eastern China. In the future, we aim to extend the proposed method to study bird migration routes in the central and western regions of China. By doing so, together with the poultry distribution among different provinces/municipalities, the relative infection risk of each province in China can be estimated accordingly. Moreover, we can also extend our model to infer infection risks at the city level by collecting more detailed data about wet lands, temperature, and poultry production and consumption, and so on.

## Conclusion

By characterizing the geographic patterns of bird migration and poultry distribution in eastern China, we have outlined three types of potential risk of human infection for different regions from February 4, 2013 to April 28, 2013. The results demonstrated in this study can provide new tools for public health policy makers to perform the active surveillance and control of H7N9 infections.

## Endnotes

^a^Source: http://sh.eastday.com/m/20121130/u1a7029021.html; http://news.sina.com.cn/o/2013-02-27/074026371385.shtml

^b^Source: http://henan.china.com.cn/news/china/201304/ 278532HXCZ.html; http://hebei.sina.com.cn/news/s/2013- 04-10/110844208.html; http://www.ha.chinanews.com/html/ yiliaoweisheng2012/dajiankang/2013/0416/15394.html

^c^Source: http://ent.xinmin.cn/2013/04/13/19712929.html; http://news.sina.com.cn/c/2013-04-13/184526817748.shtml; http://kx.zxxk.com/Article/238489.html

^d^Source: http://epaper.hilizi.com/shtml/bdcb/20130413/ 23965.shtml; http://liaoning.nen.com.cn/system/2013/04/ 11/010314017.shtml; http://www.dongbeiya.org/ShowArticle. aspx?ID=4127

## Competing interests

The authors declare that they have no competing interests.

## Authors’contributions

JL and XZ conceptualized the paper, BS and SX prepared the first draft, and GY, JL, and XZ participated in the drafting and editing of the paper. All authors read and approved the final manuscript.

## Supplementary Material

Additional file 1 Multilingual abstracts in the six official working languages of the United Nations. Click here for file
