# Supplementary material for: Inferring the potential risks of H7N9 infection by spatiotemporally characterizing bird migration and poultry distribution in eastern China
Source: Infect Dis Poverty. 2013 May 3;2:8. doi: 10.1186/2049-9957-2-8 (PMC3710089; doi:10.1186/2049-9957-2-8)
Supplement: Additional file 1 — Multilingual abstracts in the six official working languages of the United Nations. [file 2049-9957-2-8-S1.pdf]

Translation of the abstract into the six official working languages of the United Nations

استنتـــاج المخــاطر المحتملة من انتقــال الــعدوى بـفيروس H7N9 عن طريق التتبع المكاني وتوزيع هجرة الطيور والدواجن في وسط جمهورية الصين

تشي، بن يون، شانغ شيا، وجوه يينغ يانغ، وزيو تشيا يونج وليو جي مينج

الملخـص

الخلفيـــة: بالنظر إلى الانتشار الجغرافي السريع وإلى زيادة عدد الحالات المؤكدة المُصابة بالانفلونزا حديثة النوع A(H7N9)، وهي عدوى فيروسية في وسط جمهورية الصين. ونحن بصدد تطور نموذجاً توصيفياً يعتمد على نظرية التتبع المكاني لآثار هجرة الطيور وتوزيع الدواجن على انتشارها الجغرافي لعدوي H7N9.☒

الطـرق والأســاليب: تم تقدير ثلاثة أنواع من مخاطر العدوى وذلك على مدار إثنى عشر أسبوعاً، من 4 فبراير إلى 28 ابريل من عام 2013، والتي تضمنت التالي: (أولا) المخاطر الناجمة عن هجرة الطيور، (ثانياً) المخاطر الناجمة عن توزيع الدواجن، (ثالثا) الأخطار المتكاملة والناجمة عن هجرة الطيور وعلى توزيع الدواجن على حد السواء. ومن أجل تحقيق ذلك، كان علينا أولاً تطوير طريقة تقدير احتمالات هجرة الطيور بناء على البيانات البيئية والأرصاد الجوية المتاحة. ومن ثم، اعتمادنا نموذجاً حسابياً حركياً لتقدير توزيع الدواجن على أساس الإنتاج السنوي للدواجن واستهلاك كل محافظة أو منطقة/بلدية. وأخيرا، تم إنشاء خرائط المخاطر الزمانية المكانية على أساس التأثير المتكامل الناتج عن كل من هجرة الطيور وعن توزيع الدواجن. ☒

النتـــائج: وفي الدراسة يتم تقدير المخاطر الناجمة عن هجرة الطيور، حيث قُدرت مصفوفة احتمال والتي تعتمد على درجات الحرارة في فترة سبعة أيام، من يوم 4 فبراير إلى يوم 28 ابريل من عام 2013. ومن ثم كان واضحاً أن الطيور المهاجرة المقدرة في المحافظات الجنوبية الشرقية من تشي جيانج وشانغهاي وجيانجسو ظهرت أساسا من الأسبوع الأول وحتى الأسبوع الرابع، وأيضا في الأسبوع السادس، وتبعها ظهور الطيور المهاجرة في المحافظات الشرقية والوسطى من شاندونج وهيبى وبكين وتيانجين من الأسبوع السابع وحتى الأسبوع التاسع. وفي النهاية ظهرت الطيور المهاجرة أخيرا في المحافظات الشمالية الشرقية من لياونينج وجيليين وهيلونغجيانج وذلك من الأسبوع العاشر وحتى الأسبوع الثاني عشر. ☒

وفي أثناء دراسة المخاطر الناجمة عن توزيع الدواجن، تم إنشاء مصفوفة توزيع الدواجن لإبراز احتمال توزيع الدواجن. وعلى الرغم من الحقيقة أن الغالبية العظمى للعدوى الأولية قد تم الإبلاغ عنها فى إقليم شانغهاى وفي مقاطعة جيانجسو، إلا أن الخطر النسبي للإصابة بعدوى فيروس H7N9 تم تقديره استناداً على أن النموذج المتوقع لتوزيع الدواجن في جيانجسو من المحتمل أن يكون أعلى نسبيا إذا أخذنا بعين الاعتبار نظرية احتمال توزيع الدواجن.

كما أنه أثناء دراسة المخاطر المتكاملة والتي سببتها كل من هجرة الطيور وتوزيع الدواجن، تم تسجيل أعلى نسبة خطورة في خلال الفترة من الأسبوع الثامن وحتى الأسبوع الثاني عشر في المحافظات الجنوبية. وكذلك ظهرت في المحافظات الشرقية والوسطى في خلال نفس الفترة من الأسبوع الثامن وحتى الأسبوع الثاني عشر. أما في المحافظات الشمالية الشرقية فقد ظهر الخطر في الأسبوع الثاني عشر. ولذلك، يُعد تنظيم أسواق الدواجن ضرورة حتمية طالما لا يزال فيروس العدوى ينتقل بين الدواجن بطريقة غير مفهومة جيدا.

الخلاصة: وبالإشارة إلى حالات العدوى المسجلة، فقد أظهرت نتائج تتبع وتحديد المخاطر أهمية تقديم التوجيه والإرشاد للرصد النشيط وللسيطرة على فيروس H7N9 والحيلولة لعدم حدوث أية إصابات بشرية من خلال التدخل المكثف في أسواق الدواجن.

Translated from English version into Arabic by Freelance Abunar, through

TRANSLATORS WITHOUT BORDERS

应用中国大陆地区候鸟迁移和禽类分布的时空分析方法估算H7N9禽流感潜在高危区域

Benyun Shi, Shang Xia, Guo-Jing Yang, Xiao-Nong Zhou and Jiming Liu

摘要

引言：基于中国大陆地区新型禽流感病毒(H7N9)的确诊病例增多并扩散明显，我们研发了一个扩散模型用于时空分析候鸟迁移和禽类分布对禽流感H7N9病毒感染的区域扩散的影响。

方法：对2013年2月4日至4月28日共12周期间内的三种危险性进行了分析，包括一是由于候鸟迁移引起的危险性，二是由于禽类分布引起的危险性，三是综合前二者的危险性。为了评估这三类危险性，我们首先根据可获得的环境与温度资料进行了候鸟迁移可能性分析，然后根据中国各省禽类产量与消耗量资料以计算机迁移模型来估算禽类的分布。最后画出同时基于候鸟迁移和禽类分布的高危地图。

结果：基于2013年2月4日至4月28日之间每周温度的变化， 我们分析了候鸟迁移的可能性及其引起的危险性。我们发现，在第1至4周及第6周，东南部的浙江省、上海市和江苏省的候鸟停留较多，然后在第7至9周候鸟迁移至中东部的山东、河北、北京、天津，最后在第10至12周候鸟迁移至东北部的辽宁、吉林和黑龙江省。

建立了禽类概率分布的数据库用于研究禽类分布引起的危险性。尽管上海和江苏的病例报告最早，但如果仅基于禽类概率分布的话，禽类分布模型分析结果显示江苏省的H7N9病毒感染起源可能性大于上海、浙江两省。

综合分析候鸟迁移和禽类分布对禽流感病毒(H7N9)扩散的危险性结果发现，在早期的8周中东南部三省为传播高危，第8至12周在中东部的四省为传播高危，自12周起高危移到东北三省。所以，只要禽传禽的禽流感H7N9病毒传播模式不清楚时，对禽类市场的控制措施极为必要。

结论：根据已报告的禽流感病例信息，建立的禽流感传播高危地图将为主动监测与应对H7N9禽流感病毒的人群感染、强化禽类市场控制措施提供依据。

Translated from English version into Chinese by Xiao-Nong Zhou

Déterminer les risques potentiels d'infection au virus de la grippe aviaire (H7N9) en caractérisant de manière spatiotemporelle la migration d'oiseaux et la distribution de volailles en Chine continentale

Benyun Shi, Shang Xia, Guo-Jing Yang, Xiao-Nong Zhou et Jiming Liu

Abrégé

Contexte : Compte tenu de la propagation géographique rapide et du nombre croissant des cas confirmés d'infection au nouveau virus de grippe aviaire (H7N9) en Chine continentale, nous élaborons un modèle de diffusion pour caractériser de manière spatiotemporelle les impacts de la migration d'oiseaux et de la distribution de volailles sur la propagation géographique de l'infection.

Méthodes : Trois types de risques d'infection ont été évalués pendant 12 semaines, du 4 février au 28 avril 2013, dont (i) le risque dû à la migration d'oiseaux, (ii) le risque dû à la distribution de volailles et (iii) le risque cumulé dû à la fois à la migration d'oiseaux et la distribution de volailles. À cet effet, nous développons en premier lieu une méthode pour estimer la probabilité de migration d'oiseaux en fonction de données environnementales et météorologiques disponibles. Nous adoptons ensuite un modèle de mobilité informatique pour estimer la distribution de volailles en fonction de la production et la consommation annuelles de chaque province/commune. Enfin, nous créons des cartes de risques spatiotemporels selon l'impact cumulé de la migration d'oiseaux et la distribution de volailles.

Résultats : Dans l'étude d'évaluation des risques dus à la migration d'oiseaux, la matrice de probabilité a été estimée sur la base de la température sur 7 jours, du 4 février au 28 avril 2013. Il a été découvert que les oiseaux migrateurs probables apparaissent principalement dans les provinces du sud-est de Zhejiang, Shanghai et Jiangsu pendant les semaines 1 à 4 et la semaine 6, puis dans les provinces du centre-est de Shandong, Hebei, Beijing et Tianjin pendant les semaines 7 à 9, enfin dans les provinces du nord-est de Liaoning, Jilin et Heilongjiang pendant les semaines 10 à 12.

Dans l'étude d'évaluation des risques dus à la distribution de volailles, la matrice a été créée pour montrer la probabilité de la distribution. Malgré le fait que la majorité des infections initiales soient déclarées dans les provinces de Shanghai et Jiangsu, le risque relatif d'infection H7N9 estimé selon le modèle de distribution de volailles indique que la province de Jiangsu peut présenter une probabilité légèrement supérieure d'infection H7N9 que celle de Zhejiang ou Shanghai, si nous ne considérons que la probabilité de distribution de volailles.

Dans l'étude du risque cumulé dû à la fois à la migration d'oiseaux et la distribution de volailles,

le risque supérieur dans les provinces du sud-est est apparu pendant les huit premières semaines, puis dans les provinces du centre-est pendant les semaines 8 à 12, enfin dans les provinces du nord-est depuis la semaine 12. Il s'avère donc nécessaire de réguler les marchés des volailles tant que la transmission entre volailles n'est pas bien comprise.

Conclusion : En ce qui concerne les cas d'infection signalés, les résultats de la cartographie du risque établi va fournir un cadre directeur pour la surveillance active et le contrôle des infections H7N9 humaines en prenant des mesures d'intervention intensive sur les marchés des volailles.

Translated from English version into French by Christophe Legallais, through

TRANSLATORS
WITHOUT BORDERS

Оценка потенциальных рисков заражения инфекцией H7N9 на основе пространственно-временных характеристик миграции диких птиц и распределения домашней птицы на материковой части Китая

Беньюн Ши, Шанг Ксиа, Гуо-Джинг Янг, Ксяо-Нонг Джу и Джиминг Лью

Аннотация

Краткое описание: В виду быстрого географического распространения и увеличения числа подтвержденных случаев заражения новым вирусом гриппа A(H7N9) на материковой части Китая мы разработали диффузионную модель для выявления пространственно-временного влияния миграции диких птиц и распределения домашней птицы на географическое распространение инфекции H7N9.

Методы: В течение 12 недель с 4 февраля по 28 апреля 2013 года были выявлены три типа рисков заражения, включая (i) риск, связанный с миграцией диких птиц; (ii) риск, связанный с распределением домашней птицы; и (iii) совокупный риск, связанный с миграцией диких птиц и распределением домашней птицы. Для этого мы сначала разработали метод для оценки вероятности миграции диких птиц на основании имеющихся данных об окружающей среде и метеорологических данных. Затем, используя численную модель мобильности, мы оценили распределение домашней птицы на основании данных о годовом производстве домашней птицы и её потреблении в каждой провинции/муниципалитете. После этого были созданы карты пространственно-временных рисков на основании совокупного влияния миграции диких птиц и распределения домашней птицы.

Результаты: При изучении оценки риска, связанного с миграцией диких птиц, с 4 февраля по 28 апреля 2013 года была создана матрица вероятности на основе 7-дневной температуры. Было установлено, что в период с 1-ой недели по 4-ую и в течение 6-ой недели мигрирующие птицы главным образом появляются в юго-восточных провинциях Чжэцзян, Шанхай и Цзянсу. За этим следует их появление в центрально-восточных провинциях Шаньдун, Хэбэй, Бейджин и Тяньцзинь в период с 7-ой недели по 9-ую, и наконец в северо-восточных провинциях Ляонин, Гирин и Хейлунцзян в период с 10-ой недели по 12-ую.

При изучении оценки риска, связанного с распределением домашней птицы, была создана матрица, показывающая вероятность распределения. Несмотря на то, что большинство случаев начального заражения было зарегистрировано в провинциях Шанхай и Цзянсу, оценка относительного риска заражения H7N9 на основании модели распределения

домашней птицы показывает, что вероятность заражения H7N9 в Цзянсу может быть чуть выше, чем в Чжэцзяне и Шанхае, если принять во внимание распределение домашней птицы.

При изучении оценки совокупного риска, связанного с миграцией диких птиц и распределением домашней птицы, был выявлен более высокий риск заражения в первые 8 недель в юго-восточных провинциях, в центрально-восточных провинциях в период с 8-ой недели по 12-ую и в северо-восточных провинциях, начиная с 12-ой недели. Следовательно, необходимо регулировать рынки домашней птицы, так как процесс передачи вируса от одной домашней птицы к другой пока еще не очень понятен.

Заключение: С учётом зарегистрированных случаев заболевания, показанные результаты картирования рисков послужат руководством при активном наблюдении и контроле заражения людей вирусом H7N9 за счёт усиленного регулирования рынков домашней птицы.

Translated from English version into Russian by Natalia Potashnik, through

TRANSLATORS WITHOUT BORDERS

Deducción de los riesgos potenciales de la infección por H7N9 caracterizando de forma espacio-temporal la migración de pájaros y la distribución de aves de corral en China continental.

Benyun Shi , Shang Xia , Guo-Jing Yang , Xiao-Nong Zhou y Jiming Liu

Resumen

Antecedentes: En vista de la rápida propagación geográfica y el número creciente de casos confirmados de nuevas infecciones por el virus de la gripe A (H7N9) en China continental, desarrollamos un modelo de difusión para caracterizar de forma espacio-temporal los efectos de la migración de pájaros y la distribución de aves de corral en la propagación geográfica de la infección por H7N9.

Métodos: Se estimaron tres tipos de riesgos de infección durante 12 semanas, desde el 4 de febrero hasta el 28 de abril de 2013, incluyendo (i) el riesgo causado por la migración de pájaros, (ii) el riesgo causado por la distribución de aves de corral, y (iii) el riesgo integrado causado tanto por la migración de pájaros como por la distribución de aves de corral. Para lograr esto, primero desarrollamos un método para estimar la probabilidad de migración de pájaros basada en datos ambientales y meteorológicos disponibles. Después, adoptamos un modelo de movilidad computacional para estimar la distribución de aves de corral basada en la producción y consumo anual de aves de corral de cada provincia o municipio. En último lugar, los mapas de riesgos espaciotemporales fueron creados basados en el efecto integrado causado tanto por la migración de pájaros como por la distribución de aves de corral.

Resultados: En el estudio de estimación de riesgos causados por la migración de pájaros, la matriz de probabilidad fue estimada en base a la temperatura de 7 días, desde el 4 de febrero hasta el 28 de abril de 2013. Se halló que los pájaros migratorios estimados aparecen principalmente en las provincias del sudeste de Zhejiang, Shanghai y Jiangsu durante las semanas 1 a 4, y la semana 6, seguido por su aparición en las provincias centrales del este de Shandong, Hebei, Beijing y Tianjin durante las semanas 7 a 9, y finalmente aparecen en las provincias del nordeste de Liaoning, Jilin y Heilongjiang durante las semanas 10 a 12.

En el estudio de riesgo causado por la distribución de aves de corral, la matriz de distribución de aves de corral fue creada para mostrar la probabilidad de la distribución de aves de corral. Si bien el hecho de que la mayoría de las infecciones iniciales se informan en Shanghai y la provincia de Jiangsu, el riesgo relativo estimado de infección por H7N9 basado en el modelo de distribución de aves de corral predijo que es posible que Jiangsu tenga una probabilidad levemente superior de infección por H7N9 que la de Zhejiang y Shanghai, si solo tomamos en consideración la

probabilidad de distribución de aves de corral.
En el estudio de estimación de riesgos integrados causados tanto por la migración de pájaros como por la distribución de aves de corral, el riesgo mayor en las provincias del sudeste se produjo durante las primeras 8 semanas, y apareció *****en las provincias centrales del este**** durante las semanas 8 a 12, y en las provincias del nordeste desde la semana 12. Por lo tanto, es necesario regular los mercados de aves de corral mientras no se comprenda plenamente la transmisión entre aves de corral.
Conclusión: Con referencia a los casos informados sobre infección, los resultados del mapa de riesgo demostrados ofrecerán una orientación en la vigilancia y control de las infecciones humanas por H7N9 tomando intervención intensiva en los mercados de aves de corral.

Translated from English version into Spanish by two2tango, through

TRANSLATORS
WITHOUT BORDERS
